# Ultra-short laser-accelerated proton pulses have similar DNA-damaging effectiveness but produce less immediate nitroxidative stress than conventional proton beams

**DOI:** 10.1038/srep32441

**Published:** 2016-08-31

**Authors:** S. Raschke, S. Spickermann, T. Toncian, M. Swantusch, J. Boeker, U. Giesen, G. Iliakis, O. Willi, F. Boege

**Affiliations:** 1 Institute for Laser and Plasma Physics, University of Düsseldorf, Düsseldorf, Germany; 2 Institute of Clinical Chemistry and Laboratory Diagnostics, University of Düsseldorf, Medical Faculty, Germany; 3 Institute of Medical Radiation Biology, University of Duisburg-Essen, Medical School, Essen, Germany; 4 Physikalisch-Technische Bundesanstalt (PTB), Braunschweig, Germany

## Abstract

Ultra-short proton pulses originating from laser-plasma accelerators can provide instantaneous dose rates at least 10^7^-fold in excess of conventional, continuous proton beams. The impact of such extremely high proton dose rates on A549 human lung cancer cells was compared with conventionally accelerated protons and 90 keV X-rays. Between 0.2 and 2 Gy, the yield of DNA double strand breaks (foci of phosphorylated histone H2AX) was not significantly different between the two proton sources or proton irradiation and X-rays. Protein nitroxidation after 1 h judged by 3-nitrotyrosine generation was 2.5 and 5-fold higher in response to conventionally accelerated protons compared to laser-driven protons and X-rays, respectively. This difference was significant (p < 0.01) between 0.25 and 1 Gy. In conclusion, ultra-short proton pulses originating from laser-plasma accelerators have a similar DNA damaging potential as conventional proton beams, while inducing less immediate nitroxidative stress, which probably entails a distinct therapeutic potential.

High intensity laser devices are capable of delivering protons at energies sufficient for the penetration of cells and tissues. Such laser-accelerated protons (LAP) are of great practical interest for clinical health care^1^,^2^,^3^, because they may pose a reasonably priced alternative to conventionally accelerated protons (CAP) currently implemented at large centres for ion beam cancer radiotherapy. To evaluate the suitability of LAP for radiotherapy, experimental setups have been devised for the irradiation of living tumour cell cultures and tissues that allow the comparison with CAP and other types of ionising radiation by established radiobiological readouts and endpoints^4^,^5^,^6^,^7^. In summary, these studies suggest that the biological effectiveness of LAP and CAP is roughly equal with regard to DNA damage and tumour cell killing.

CAP, LAP and other ionising radiation damage the carbohydrate backbone and the bases of DNA by direct energy transfer, and generate radicals by water radiolysis that are responsible for indirect DNA damage^8^. Moreover, ionising radiation induces delayed responses involving enhanced nitroxidative stress, which is thought to contribute to late and non-targeted effects^9^,^10^,^11^,^12^. The half-life of the primary radicals induced by the interaction of protons with H_2_O, O_2_ or NO**·** is in the ns range^9^,^13^,^14^. LAP are delivered at instantaneous dose rates of around 10^8^ Gy/s, while CAP used in this study are delivered at around 0.01 Gy/s. Thus, the delivery rate of CAP is about 11 orders of magnitude lower than the lifetime of the primary radicals thereby induced, while the temporal delivery rate of LAP is in the same order of magnitude. Consequently, LAP could possibly have a different radical generating potential and characteristics than CAP, despite having a similar DNA damaging potential. In the present study we have tested this hypothesis, comparing DNA damage and early protein oxidation inflicted by equal doses of LAP originating from a TW laser system or CAP produced by a Van de Graaff accelerator. Our results demonstrate that in LAP the balance between immediate redox effects and DNA damage is indeed shifted towards more DNA damage with less nitroxidative stress.

## Results

### LAP and CAP have similar effectiveness in inducing DNA double strand breaks

**Figure 1:**
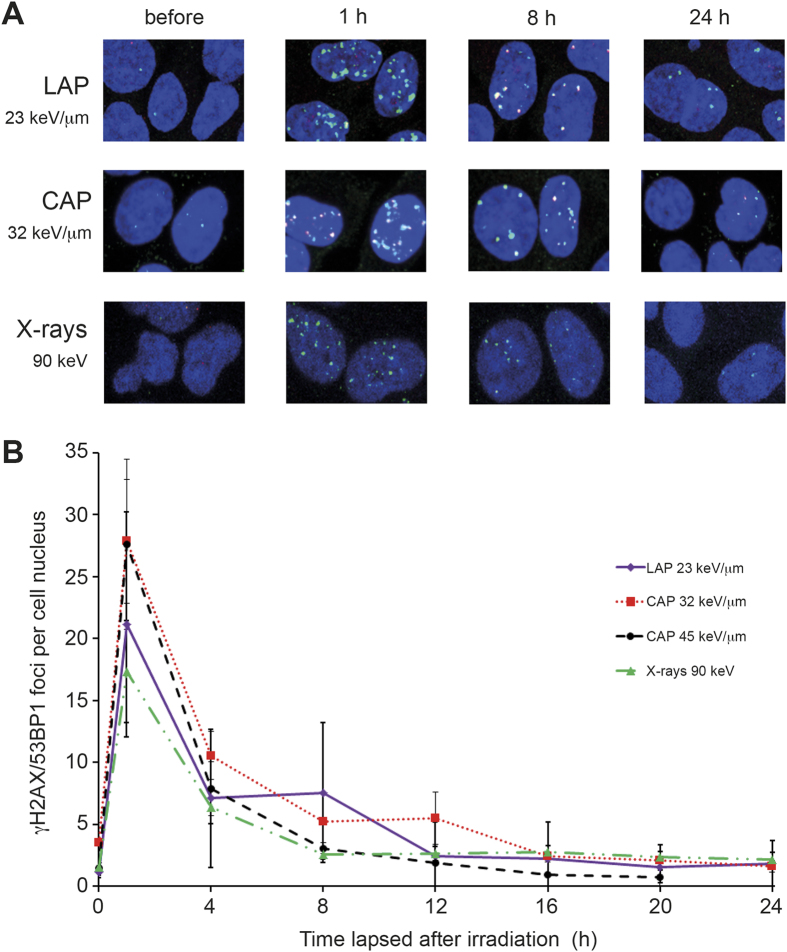
Time courses of DNA damage foci formation. (**A**) Representative confocal immune-fluorescent images of cells in mid plane at various time-points after irradiation with 1 Gy of LAP (top), 0.9 Gy of CAP (middle) or 1 Gy of 90 keV X-rays (bottom). Merged images show γH2AX (green), 53BP1 (red), DNA (blue). γH2AX/53BP1 positive foci are red and yellow. (**B**) Estimation of formation and decay of γH2AX/53BP1 foci upon irradiation with 0.9–1 Gy of the indicated radiation modality. Each time point represents the mean of three independent experiments and in each experiment three microscopic fields were analysed and counted. Data given as mean ± SD.

As a first step, we compared the time courses of cellular responses to DNA double strand breaks (DSBs) induced by LAP, CAP or X-rays, using histone H2AX phosphorylated at Ser 139 (γH2AX) and the damage recognition protein 53BP1 as markers. Each single marker has been firmly established for detecting DSBs^15^,^16^,^17^,^18^. Here, their simultaneous use ascertained a maximum of sensitivity and specificity. Cellular density of γH2AX/53BP1- double positive foci was measured by automated quantitative high content confocal laser-scanning microscopy of cells fixed and immune-stained at various time points following irradiation with equal doses (between 0.64 and 1 Gy) of LAP, CAP or 90 keV X-rays. Figure 1A shows representative images of the cells, while Fig. 1B shows time-courses of the nuclear density of γH2AX/53PB1 foci determined by computer-aided quantitative analysis of corresponding z-stacks. It can be easily noticed that the time-courses of decay of γH2AX/53BP1 foci were similar for all types of radiation tested. Regardless of the irradiation type, cells exhibited 19–27 foci at one hour after irradiation. The number of foci decreased gradually until a constant base line of 2–4 foci per cell was reached 12–16 h later. The base line post irradiation was similar to the average prevalence of foci before irradiation (i.e. at time zero). These observations suggest that LAP and CAP have a similar effectiveness to induce DNA double strand breaks.

**Figure 2:**
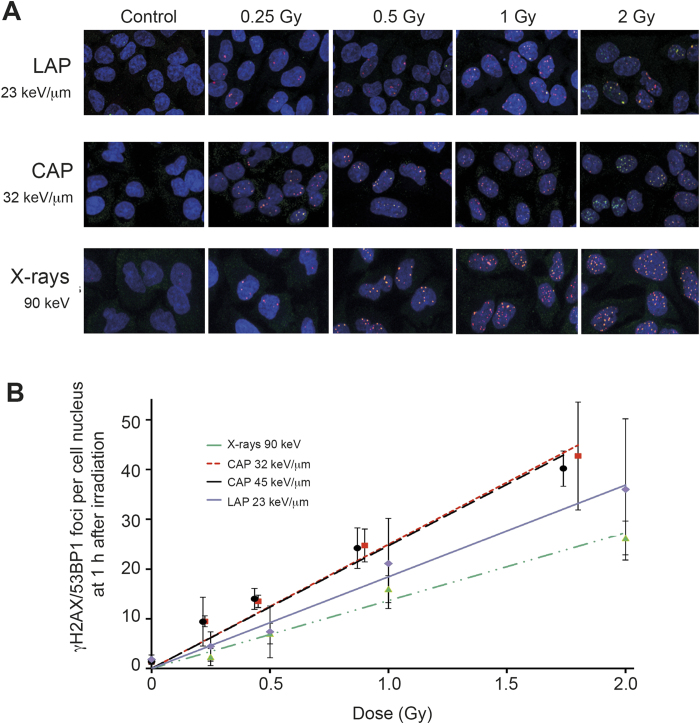
Dose responses of DNA damage foci formation. (**A**) Representative confocal immune-fluorescent images of cells in mid plane obtained 1 h after exposure to indicated doses of LAP (top), CAP (middle) or 90 keV X-rays (bottom). The negative control (0 Gy) was sham-irradiated. Merged images show γH2AX (green), 53BP1 (red), and DNA (blue). γH2AX/53BP1 positive foci are red-yellow. (**B**) Number of γH2AX/53BP1 foci induced by various radiation modalities. Each data point represents the mean of three independent experiments. In each experiment three microscopic fields were analysed and averaged. Data corrected for background are given as mean ± SD. Plotted lines represent results of linear regression (Pearson’s coefficient R^2^ > 0.95 and p < 0.001 throughout).

To follow up on this notion, dose responses of yield of γH2AX/53BP1 foci at 1 h after irradiation were compared between LAP and CAP. For this purpose, a dose range from 0 to 2 Gy was studied at an average initial energy of 2.1 MeV and an averaged linear energy transfer (LET) of 23 keV/μm for LAP and 32 keV/μm and 45 keV/μm for CAP. 90 keV X-rays served as reference. Figure 2A shows representative mid plane images of A549 cells subjected to immune-staining and confocal immunofluorescence microscopy 1 h after exposure to the various doses of LAP, CAP or 90 keV X-rays. A quantitative estimation of the nuclear density of γH2AX/53PB1 foci obtained by automated computer aided analysis of corresponding z-stacks is summarised in Fig. 2B. A linear relationship between applied dose and the number of induced γH2AX/53BP1 foci was confirmed by linear regression analysis of the background-corrected data. The relative biological effectiveness (RBE) derived from the data in Fig. 2B was 1.8 ± 0.8 for CAP with LETs of 32 and 45 keV/μm and 1.4 ± 1.0 for LAP with an LET of 23 keV/μm (Of note: The LET value of LAP was calculated considering an energy spread between 0 and 2.2 MeV within the cell layer and not from a single energy). RBE-calculations from these data seem meaningless, since the yield of γH2AX/53BP1 foci across the investigated dose range was not significantly different between LAP, the two LET-varieties of CAP and X-rays (p-values obtained by the two-tailed, unpaired t-test or Mann Witney U-test were >0.1 in all comparisons). In summary, these data indicate that the mode of ion acceleration does not significantly affect the cellular response in terms of DSB induction, at least not within the energy range studied here.

### Induction of nitroxidative stress differs between exposures to LAP and CAP

**Figure 3:**
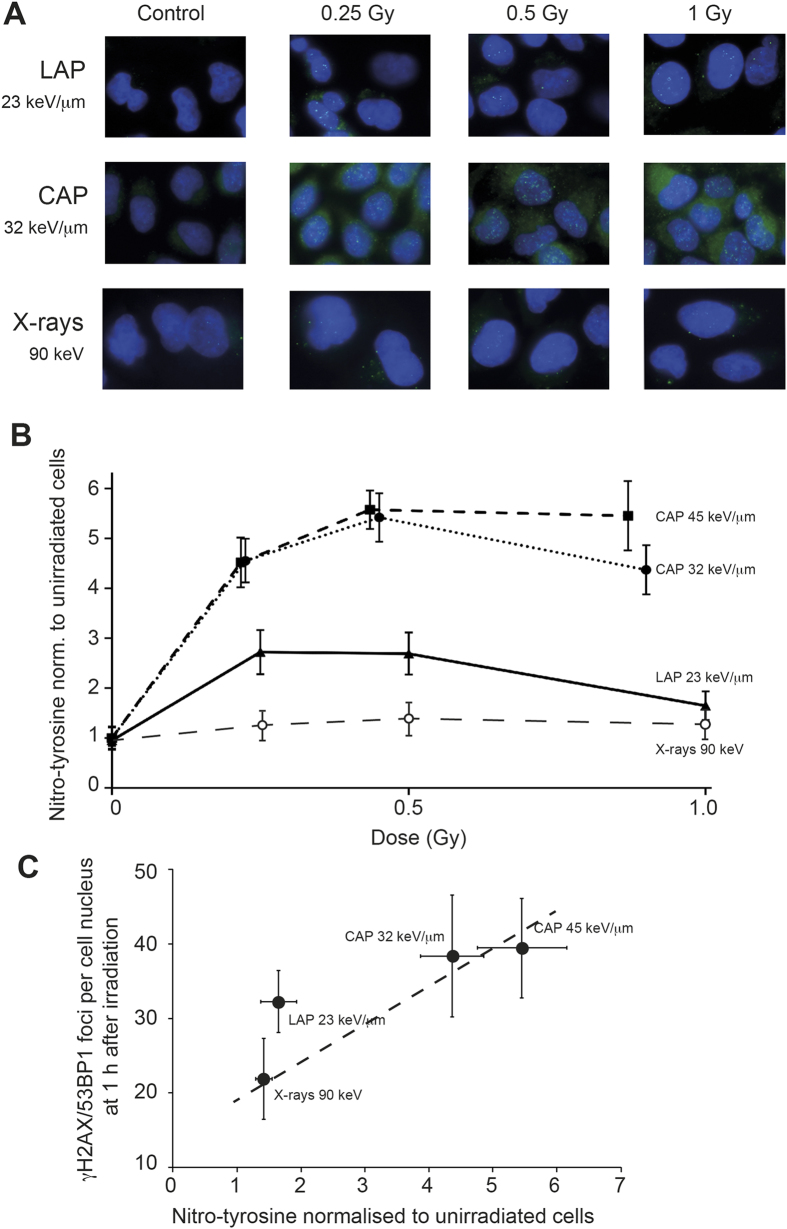
Induction of 3-nitrotyrosine. (**A**) Representative epi-fluorescence images of cells in mid plane obtained 1 h after exposure to indicated doses of LAP (top), CAP (middle) or 90 keV X-rays (bottom). Merged images show 3-nitrotyrosine (green) and DNA (blue). (**B**) Quantitative estimation of cytosolic 3-nitrotyrosine staining expressed as radiation-induced increase above background at 0 Gy. Each data point represents the mean of three independent experiments and in each experiment three microscopic fields were analysed and averaged. Data given as mean ± SD. Data above 0 Gy were significantly different between CAP 32 keV/μm and LAP 23 keV/μm (p < 0.05, Wilcoxon’s Test for unpaired samples). (**C**) DNA-damage foci formation 1 h after exposure to maximal dose of the indicated irradiation modality (data from Fig. 1B) plotted as a function of corresponding values for induction of 3-nitrotyrosine (data from Fig. 3B). Dotted line: linear regression of CAP and X-ray values, Pearson’s coefficient R^2^ = 0.52 (p < 0.01).

To address the question, whether LAP and CAP have a different effectiveness to generate nitroxidative stress, we used 3-nitrotyrosine, which is an established marker for protein nitroxidation by peroxynitrite and other radicals^19^. A549 cells were exposed to various doses of LAP, CAP or 90 keV X-rays. 1 h later, the amount of cell-bound 3-nitrotyrosine was determined by immune-cytochemistry. Representative epi-fluorescence images (Fig. 3A) clearly indicate that CAP induced higher cellular levels of 3-nitrotyrosine (shown in green) than LAP or X-rays. For a quantitative estimation of the difference the extent of radiation-induced 3-nitrotyrosine formation was calculated as fold increase of 3-nitrotyrosine specific mean fluorescence intensity over background determined in sham-irradiated controls. Figure 3B shows the values as a function of radiation dose. Following irradiation with CAP, the 3-nitrotyrosine label strongly increased with dose until 0.5 Gy and remained at this level at higher doses. CAP with LETs of 32 or 45 keV/μm had similar effects (the slight difference at the highest dose was not significant), which conforms to the similar DSB induction demonstrated in Fig. 2B. Across the entire dose range investigated, the effectiveness of X-rays to induce 3-nitrotyrosine was at least 5-fold lower than that of CAP. Most notably, the induction of 3-nitrotyrosine by LAP was also 2–3-fold lower than that of CAP. Indeed, at doses around 1 Gy the effectiveness of LAP with an LET = 23 keV/μm to induce 3-nitrotyrosine was not significantly different from 90 keV X-rays, whereas the effectiveness to induce DSB was not significantly different from CAP with an LET of 32 or 45 keV/μm (Fig. 3C).

## Discussion

The salient finding of this study is that LAP and CAP have a similar effectiveness to induce DSB, while LAP have a far lower potential than CAP to induce nitroxidative stress leading to immediate tyrosine-nitration. Indeed the effectiveness of LAP to induce this well established cellular endpoint of nitroxidative stress seems to be more similar to low LET ionising radiation such as 90 keV X-rays, whereas the effectiveness to induce DSB is in the same range as that of protons generated by continuous acceleration. These hybrid properties of LAP may provide unique opportunities for human cancer therapy.

High-energy protons induce DNA damage in the form of DSB, single-stranded DNA breaks (SSB), DNA base damage and clusters thereof (complex DSB)^20^. DNA damage is either induced by direct energy deposition to the 2-deoxyribose moiety or the bases of DNA (direct effect), or by water radiolysis generating radicals, which then interact with DNA (indirect effect)^8^. The biological effectiveness of protons is mostly a function of the frequency and complexity of the induced DNA breaks^20^,^21^, which in turn is a function of LET. All available studies report that LAP and CAP have a similar RBE in terms of DSB induction and cell killing^4^,^5^,^6^,^7^,^22^. However, the induction of direct DNA damage versus nitrosoxic stress has not been compared between LAP and CAP. In fact, it is generally assumed that the two types of protons have exactly the same biological effects at all endpoints^4^,^5^,^6^,^7^,^22^. Our study provides a first indication that this may not be the case.

**Figure 4:**
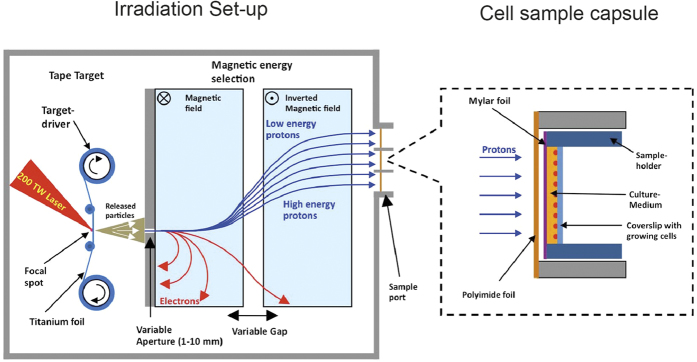
Experimental setup for cell irradiation with LAP. The beam of a 200 TW Arcturus laser was focused onto an automatically rotating tape target. Energy selection inside the vacuum chamber was performed using a magnetic double yoke. At the exit point of the chamber a polyimide foil sealed the vacuum against the normal atmospheric pressure. Onto that seal were mounted the sample ports housing the cell sample capsule or proton-detection-devices such as CR-39 nuclear track detectors and image plates.

The similar DNA damaging potential of LAP and CAP observed here is in good agreement with previous studies^4^,^5^,^6^,^7^,^22^. However, in absolute terms, the RBE values determined by us and those reported previously diverge to some extent, which is most probably due to differences in reference X-ray energies, experimental endpoints, and the different energy averages of LAP proton spectra. The kinetics of disappearance or decay of DNA damage foci observed here in response to LAP and CAP are similar to those recently obtained with other radiation modalities^23^ and can be considered typical for ionising radiation^24^. Similarly, the linearity of the dose response curve of LAP reported here, suggests that DSBs were formed as a result of single ion interactions, as is typical for proton irradiation^8^,^25^. One previous study^7^ reported a non-linear DSB response of γH2AX foci formation to LAP at doses of 3 and 7 Gy. This divergence is probably due to the higher doses and different experimental set up used in that study, which encompassed significant time lapses between sequential laser shots required for dose escalation. Since dephosphorylation of gammaH2AX by nucleosome-associated phosphatases starts almost immediately after DSB induction^26^,^27^,^28^,^29^, it may cause loss of linearity of γH2AX yield in this experimental setup. In our study, the time lapse between sequential laser shots was only 3 s (due to the use of a rotating tape target, see Fig. 4), implying a similar influence of dephosphorylation on γH2AX yield at all doses tested.

Reactive radicals induced by ionising radiation not only contribute to DNA damage^30^, but have other physiological effects. Delayed and persistent radical stress caused by signaling between irradiated cells and unirradiated bystanders is thought to contribute to late cellular effects of irradiation including cell killing (so called bystander effect)^9^,^10^,^11^,^12^. Here, we focus on immediate nitroxidative stress most probably due to water radiolysis, which generates primary radicals with a lifetime in the ns range. These include solvated electrons (e_aq_^−^), hydroxyl radicals (**·**OH), hydrogen atoms (**·**H) and hydroperoxide radicals (HO_2_**·**). In the presence of molecular oxygen e_aq_^−^ and **·**H are converted into superoxide radicals (O_2_**·**^−^) and HO_2_**·** respectively, which at neutral pH are in a dynamic equilibrium with a preponderant shift towards O_2_**·**^−^. Decay of these radicals causes the formation of the longer lived molecule hydrogen peroxide (H_2_O_2_)^31^. H_2_O_2_ yield increases with LET^32^, due to inter-track recombination of radicals and multiple ionisation events^33^,^34^, but appears to be independent of dose rate^35^. However, for high LET particles, the yields of H_2_O_2_ for the same LET but for different particles can be very different, suggesting that LET alone may not be sufficient to correctly characterise the energy deposition structure^36^. Indeed it has been demonstrated by fast spectroscopy, that the yield of short lived radicals (e_aq_^−^, **·**OH, **·**H, HO_2_**·**) in response to water irradiation with pulsed heavy ions decreases with increasing LET^13^,^14^. In analogy, it seems conceivable that water radiolysis by pulsed protons (as performed here) similarly results in a lower yield of shortlived radicals, thus causing the lower tyrosine nitration observed. In other words, the lower tyrosine nitration possibly reflects an altered balance between direct DNA damage and indirect effects mediated by radicals emerging form water radiolysis, most notably **·**OH. This would imply that indirect DNA damaging effects (e.g. DNA base oxidation) should also be lower in the case of LAP, which was however, not directly addressed in this paper. Moreover, the mechanism of tyrosine nitration in response to pulsed proton irradiation remains unclear.

The established route for tyrosine nitration is the reaction with peroxynitrite-derived radicals^19^. Protonation of peroxynitrite and the subsequent decomposition of the resulting peroxynitrous acid into **·**OH and NO_2_^−^ allows **·**OH-mediated oxidation of tyrosine, giving rise to an oxidising tyrosyl radical able to react with NO_2_^−^ to form 3-nitrotyrosine^37^. A more likely alternative implicates the reaction of peroxynitrite with carbon dioxide to form nitrosoperoxycarbonate, the decomposition of which leads to the formation of NO_2_^−^ and the highly reactive carbonate anion radical (CO_3_^−^**·**) that is capable of one-electron oxidation of tyrosine. Recombination of NO_2_^−^ and the neutral oxidising radical that arises from the deprotonation of the tyrosine radical gives rise to 3-nitrotyrosine^19^. However, peroxynitrite is not considered a major downstream intermediate of the initial action of ionising radiation including accelerated protons, which mainly causes the generation of H_2_O_2_^31^. In contrast, peroxynitrite results from the reaction of O_2_**·**^−^ with nitric oxide (NO**·**)^38^, which is more likely part of the delayed radiation response that mostly affects cells which have not been directly hit by a radical event (i.e. the bystander effect)^9^,^10^,^11^,^12^. However, the bystander effect cannot have contributed much to the differences in tyrosine nitration observed here, since at the doses investigated, on average, all cells received more than one hit, and as a result, there were very few bystander cells present in the experiments (see Figs 1A and 2A).

However, several pathways can be envisioned by which the immediate redox effects of ionising radiation (i.e. water radiolysis) may directly lead to 3-nitrotyrosine formation if NO**·** is present. This is the case in A459 cells, which constitutively express nitric oxide synthase, and, upon culture in media containing NO**·** donors (as done here), generate internally a significant amount of the NO**·**^39^. Endogenously produced NO**·** persists in the cells as NO_2_^−^ or nitrosothiols^40^. Peroxynitrite can be generated via the reaction of nitrosothiols with H_2_O_2_ (emerging from water radiolysis)^41^ and tyrosine nitration could subsequently occur via the established reaction with peroxynitrite-derived radicals^19^. However the production of NO_2_**·** *via* the oxidation of nitrosothiols by H_2_O_2_ is likely to be in most cases a delayed reaction that would prevent any efficient recombination with the short-life tyrosyl radical. Alternatively, tyrosine could be nitrated directly by radicals generated by the decomposition of cellular NO_2_^−^ 42, which is highly probable at conditions allowing water radiolysis, since the O-N-O bond energy (93.3 KJ mol^−1^ ≈ 0.93 eV) is approximately five time lower than that of O-H in water (450 KJ mol^−1^ ≈ 4.5 eV)^43^,^44^,^45^. However, generation of 3-nitrotyrosine by this reaction would require that both tyrosyl and NO_2_**·** radicals are generated in a very close vicinity, which seems a rather unlikely event. Finally, cellular NO**·** has been shown to react with tyrosyl radicals (generated by **·**OH-mediated oxidation of tyrosine), which in the presence of H_2_O_2_ yields 3-nitrotyrosine^46^. However, the necessity of two successive reactions is not in favour of an immediate generation of 3-nitrotyrosine with high yield along this pathway.

While the exact reactive cascade linking the primary radical generation of ionising radiation to the formation of 3-nitrotyrosine remains obscure, our data clearly show that it occurs as there exists a covariance of irradiation dose, DSB-induction and tyrosin-nitration - which incidentally is linear for X-rays and CAP (Fig. 3C). Interestingly, LAP are an outlier in that correlation in as much as they produce significantly less nitroxidative stress relative to DSBs. The most likely explanation for this unique property of LAP is their excessively high dose rate. It appears as if redox chemistry changes when dose deposition occurs as an ultra-short pulse, with similar duration as the lifetime of the primary radicals thereby generated (as is the case with LAP).

Despite the uncertainty about the precise redox chemistry involved, our data strongly indicate that LAP exhibit a much lower effectiveness for inducing immediate nitroxidative stress than CAP. The shift in the balance between redox effects and DSB induction towards more DSB induction shown here with LAP may affect DNA damage by indirect effects, as well as protein oxidation. Such shifts could be beneficial or adverse with respect to cancer therapy, and future putative clinical applications will have to take into account these unique properties of LAP as compared to CAP.

## Methods

### Application of LAP

LAP were generated in single shot mode using the 200 TW ARCTURUS laser system at the University of Düsseldorf/Germany focused on a titanium foil target. Emerging proton radiation was directed at monolayers of living cells in a similar fashion as described^4^,^5^,^6^,^7^,^22^. Cells grown on coverslips were exposed to LAP at initial beam energy of 2.1 MeV +/− 0.25 MeV. Magnetic selection of the beam emerging from the laser-irradiated titanium foil resulted in a position-dependent spread of proton energies across the irradiated cell layer ranging from 0 and 2.2 MeV with an average LET of 23 keV/μm. This energy range was chosen because the Bragg peak is located close to the cell mid-plane for a significant part of the spectrum. Distinct doses between 0.25 and 2 Gy were created by the cumulative application of several laser shots with a known dose per shot (evaluated from a typical laser shot). Dose uncertainty was estimated to be 23%, based on the relative uncertainty of the typical shot evaluated at 64% and the minimum number of shots during an irradiation procedure, which was eight. The time lapse between shots was 3 s. The given uncertainty takes into account the effect of different energies in the spectrum and their respective penetration depths considering the specific setup of layers for this experiment.

Cells to be irradiated were enclosed in a ring-shaped sample holder sealed on one side with a 1.5 μm thick Mylar foil and on the other side with a glass slide. The cells to be irradiated were grown on the glass slide with the cell layer facing towards the Mylar foil. The interspace between the Mylar foil and the cell-bearing surface of the glass slide was 18 μm. The sample holder was mounted on a 25 μm polyimide foil window with a diameter of 2 cm serving as vacuum seal. Protons reached the cells after penetrating the polyimide window, the Mylar foil and the medium-filled interspace between Mylar foil and cell layer of about 15 μm. Three of such ports were employed simultaneously. The middle port served for cell irradiation, while the other two ports received CR-39 nuclear track detectors and image plates for dose measurements. Figure 4 shows a schematic drawing of the entire irradiation set-up.

### Application of CAP

CAP were generated by a Van de Graaff accelerator at PTB Braunschweig, Germany as previously described^47^. In order to deliver a homogenous proton beam over the entire cell layer, the primary proton beam was scattered by a 0.5 μm thick gold foil in the center of a scattering chamber. The cells were mounted at an angle of 45° and a distance of 172 mm using the same type of cell sample capsule as for exposure to LAP. The protons underwent energy losses while passing the gold foil, the vacuum seal (5 μm Molybdenum foil) an air gap of 3 mm, the Mylar seal of the sample capsule and a layer of medium of about 15 μm. The initial beam energy of 1.54 MeV from the Van de Graaff accelerator was chosen, in order to produce an energy spectrum with a peak around 0.4 MeV and a width of about 0.13 MeV (FWHM). The average LET of the spectrum was 45 keV/μm. In addition, a higher initial energy of 1.7 MeV was utilised for generating an energy spectrum with a peak of around 0.7 MeV that resulted in an average LET of the spectrum of 32 keV/μm and a width of about 0.11 MeV (FWHM). For calculation of energy losses and resulting LET values the software package SRIM-2013 by James Ziegler was used (www.srim.org). Calculations were based on the nominal thickness of the foils and the measured thickness of the medium layer. The dose applied to the cells was calculated from the average LET-value and the proton flux, which was determined from simultaneous measurements of scattered protons at 135 degrees. The uncertainty in the dose values is estimated at about 10% and 15% for irradiations with protons of 1.7 MeV and 1.54 MeV, respectively. Sham-irradiated control samples were treated similarly without proton exposure.

### X-ray irradiation

X-rays were generated by a 320 kV cathode generator at the University of Duisburg-Essen Medical School, Essen, Germany (Isovolt 320HS, Seifert/Pantak, General Electric, Frankfurt, Germany) using a voltage of 320 kV with a 1.65 mm aluminum filter and delivering a photon energy of approximately 90 keV. Even irradiation was ensured by rotating the radiation table, which was installed in a distance of 50 cm from the X-ray source of the machine. The radiation dose was determined with a universal dose meter (UNIDOSE, PTW, Freiburg, Germany) connected to an in-field ionization monitor. Radiation dose calibration was confirmed by Fricke’s chemical dosimetry at the position for cell irradiation. The relative uncertainty for the irradiation doses is 15%.

### RBE determination

RBE was calculated from the data in Fig. 2B by comparing the slopes of linear regressions of dose responses to X-rays with corresponding slopes of CAP or LAP. Imprecision of RBE determination was calculated by first determining the relative uncertainties of the slopes of the fit for each irradiation modality by error propagation on the ratio of average foci number per nucleus over dose. Gaussian error propagation was then performed to determine the overall uncertainties of the RBE values. Tests on the significance of differences in the yield of γH2AX/53BP1 foci were performed on background-corrected data. The Mann Witney U-test or the t-test was applied according to the distribution of the data. Two tailed test formats for unpaired samples were used, since the doses related to the compared data points were not exactly equal.

### Cell culture and immunocytochemistry

A549 lung cancer cells were grown in McCoy’s 5A medium supplemented with 10% fetal bovine serum and 1% penicillin/streptomycin in 5% CO_2_ atmosphere at 37 °C. For irradiation, cells were sub-cultured on circular glass slides of 13 mm diameter to a density of about 70.000 cells per slide. 1 to 24 h after irradiation, cells were washed with pre-warmed PBS, fixed with 4% formaldehyde (10 min, 20 °C) and made permeable (15 min, 20 °C) with Triton X-100 (0.5% for γH2AX/53BP1 staining; 0.1% for 3-nitrotyrosine staining). Cells were then treated (15 min, 20 °C) with PBS containing 1% bovine serum albumin and 10% normal goat serum (blocking buffer) and subsequently incubated with primary antibodies diluted in blocking buffer for 1 h at 20 °C or 12 h at 4 °C. Primary antibodies against histone H2AX phosphorylated at Ser 139 (γH2AX) obtained from Abcam, Cambridge, UK were diluted 1:400; antibodies against the damage recognition protein 53BP1 obtained from Santa Cruz Biotechnology (Dallas, TX) were diluted 1:200; antibodies against the protein oxidation marker 3-nitrotyrosine obtained from Life Technologies, Carlsbad, CA were diluted 1:1000. Cells were counterstained (1 h, 20 °C) with fluorescently labelled secondary antibodies diluted in blocking buffer (Alexa 488, Invitrogen; 1:400 for detection of γH2AX; 1:1000 for detection of 3-nitrotyrosine; Alexa 568, Invitrogen; 1:400 for detection of 53BP1) and embedded in anti fade reagent (Invitrogen, Carlsbad, CA) containing 4′,6-diamidin-2-phenylindol (DAPI). Cells were imaged using a Leica TCS-SP5 confocal laser scanning microscope equipped with a 63x plan achromat oil immersion lens with an aperture of 1.4 coupled to a photomultiplier (Leica Systems, Wetzlar, Germany). Z-stacks spanning the thickness of a cell nucleus were recorded, with intervals between z-sections set to 500 nm, a resolution of 1024 × 1024 pixels and a pixel size of 200 nm. Foci positive for γH2AX and 53BP1 were counted using the computer software Imaris V6.0 (Oxford Instruments, Concord, MA). Fluorescent images of 3-nitrotyrosine stained cells were acquired using an inverted microscope (Axiovert100, Carl Zeiss, Jena, Germany) equipped with a digital camera (Visitron System GmbH, Munich, Germany). Mean fluorescence intensity of 3-nitrotyrosine staining was averaged over signals of single cells in at least 3 microscopic areas after background subtraction. Data were normalised to un-irradiated controls using the computer software MetaMorph (Vers.7.7.7.0, Molecular Devices, Sunnyvale, CA).

## Additional Information

**How to cite this article**: Raschke, S. *et al*. Ultra-short laser-accelerated proton pulses have similar DNA-damaging effectiveness but produce less immediate nitroxidative stress than conventional proton beams. *Sci. Rep.* **6**, 32441; doi: 10.1038/srep32441 (2016).
